# Cerebrospinal fluid osmolality cannot predict development or surgical outcome of idiopathic normal pressure hydrocephalus

**DOI:** 10.1186/s12987-022-00349-5

**Published:** 2022-06-27

**Authors:** Eva Kjer Oernbo, Annette Buur Steffensen, Hanne Gredal, Helle Harding Poulsen, Nina Rostgaard, Cecilie Holm Rasmussen, Marlene Møller-Nissen, Anja Hviid Simonsen, Steen Gregers Hasselbalch, Marianne Juhler, Nanna MacAulay

**Affiliations:** 1 Department of Neuroscience, Faculty of Health and Medical Sciences, University of Copenhagen, Blegdamsvej 3B, 2200 Copenhagen, Denmark; 2 Department of Veterinary Clinical Sciences, University of Copenhagen, Copenhagen, Denmark; 3 Department of Neurosurgery, Neuroscience Center, Copenhagen University Hospital - Rigshospitalet, Copenhagen, Denmark; 4 Danish Dementia Research Centre, Department of Neurology, Neuroscience Centre, Copenhagen University Hospital - Rigshospitalet, Copenhagen, Denmark; 5 Department of Clinical Medicine, Faculty of Health and Medical Sciences, University of Copenhagen, Copenhagen, Denmark

**Keywords:** CSF, Osmolarity, Biomarker, iNPH

## Abstract

**Background:**

The etiology of idiopathic normal pressure hydrocephalus (iNPH) is currently unknown. With no visible obstructions, altered cerebrospinal fluid (CSF) dynamics may explain the accumulation of ventricular fluid. We hypothesized that elevated osmolality in the CSF of iNPH patients could potentiate formation of ventricular fluid and thereby cause the disease progression and/or predict the surgical outcome. To address this hypothesis, we determined the lumbar and ventricular CSF osmolality of iNPH patients at different disease stages and compared with lumbar CSF samples obtained from control subjects.

**Methods:**

The osmolality of CSF was determined on a total of 35 iNPH patients at diagnosis and at the subsequent treatment with shunt surgery (n = 20) and compared with the CSF osmolality from 20 control subjects. Simultaneously collected lumbar and ventricular CSF samples from experimental pigs were used to evaluate the compatibility between CSF from different compartments.

**Results:**

We found no evidence of increased osmolality in the CSF of iNPH patients upon diagnosis or at the time of shunt treatment months after the diagnosis, compared with control individuals. CSF tapped from the lumbar space could be used as a read-out for ventricular CSF osmolality, as these were similar in both the patient group and in experimental pigs. We further observed no correlation between the CSF osmolality in iNPH patients and their responsiveness to shunt surgeries.

**Conclusions:**

The osmolality of lumbar CSF is a reliable reflection of the ventricular CSF osmolality, and is not elevated in iNPH patients. iNPH therefore does not appear to arise as a function of osmotic imbalances in the CSF system and CSF osmolality cannot serve as a biomarker for iNPH or as a predictive tool for shunt responsiveness.

## Introduction

CSF surrounds the mammalian brain and fills the central ventricular cavities. Abnormal expansion of the ventricular space signifies the pathological condition known as hydrocephalus. Although some forms of hydrocephalus arise following blockage of CSF circulation within the ventricular system and/or the CSF drainage routes, clinical hydrocephalus often manifests in the absence of a detectable blockage of these pathways. Idiopathic normal pressure hydrocephalus (iNPH) patients belong to the latter group, as these present with enlarged ventricles in the absence of a discernible blockage of the CSF pathways [1, 2]. In addition to their ventriculomegaly, this elderly patient group presents with the clinical triad of symptoms consisting of urinary incontinence, gait disturbances and cognitive decline [1, 3]. Drainage of the excess ventricular fluid by shunt insertion often improves the patients’ clinical status [4, 5], which suggests that CSF accumulation is part of the underlying etiology of iNPH. However, it remains unresolved what drives the pathological CSF accumulation. iNPH may associate with elevated CSF outflow resistance [6, 7] and/or with hyperdynamic CSF flow [8–10], which could potentially represent CSF hypersecretion. Although the osmotic gradient between CSF and plasma in healthy rats, pigs, and humans appears negligible [11], experimental elevation of CSF osmolality increases the rate of CSF secretion in experimental animals [11–14] and leads to hydrocephalus in healthy rats [15, 16]. Pathological elevation of CSF osmolality could thus precipitate the enlarged ventricles observed in iNPH patients. However, diagnostic CSF sampling is routinely obtained from the lumbar section of the spine, the composition of which may differ from that residing in the ventricular compartment [17–21]. CSF osmolality disturbances may therefore not be detected in lumbar CSF samples despite their potential occurrence in the ventricular compartment with direct access to the CSF production site at the choroid plexus.

The shunt surgery commonly used to treat iNPH patients diverts excessive ventricular fluid into the peritoneal cavity. Even though 80% of the iNPH patients appear to respond to these shunts with clinical improvement of their symptoms [22], complications, shunt failures and subsequent shunt revisions often require repeated neurosurgeries, which may be associated with serious complications [22, 23]. Therefore, a comprehensive risk–benefit analysis of each patient is crucial to decide whether surgical ICP management should be initiated. Predictive tools to select the group of patients with enhanced probability of successful treatment are greatly needed [24]. The clinical severity of the iNPH disease differs greatly. In Copenhagen, iNPH patients with more severe symptoms are often referred to neurosurgical shunt implantation, whereas patients with milder symptoms are not operated immediately, but followed for progression. This approach is based on the higher degree of diagnostic uncertainty in patients with mild symptoms, but studies suggest that early shunt surgery improves patient survival, stressing the need for early diagnostic biomarkers [25]. An early iNPH diagnosis could possibly be aided by inclusion of a biomarker, such as altered CSF osmolality if this would reflect disease severity.

We here assessed the CSF osmolality in iNPH patients versus that obtained in a control group, evaluated the resemblance between lumbar and ventricular CSF osmolality, and determined whether CSF osmolality could serve as a predictive tool to select patients benefitting from surgical management.

## Methods

### Patients

**Fig. 1:**
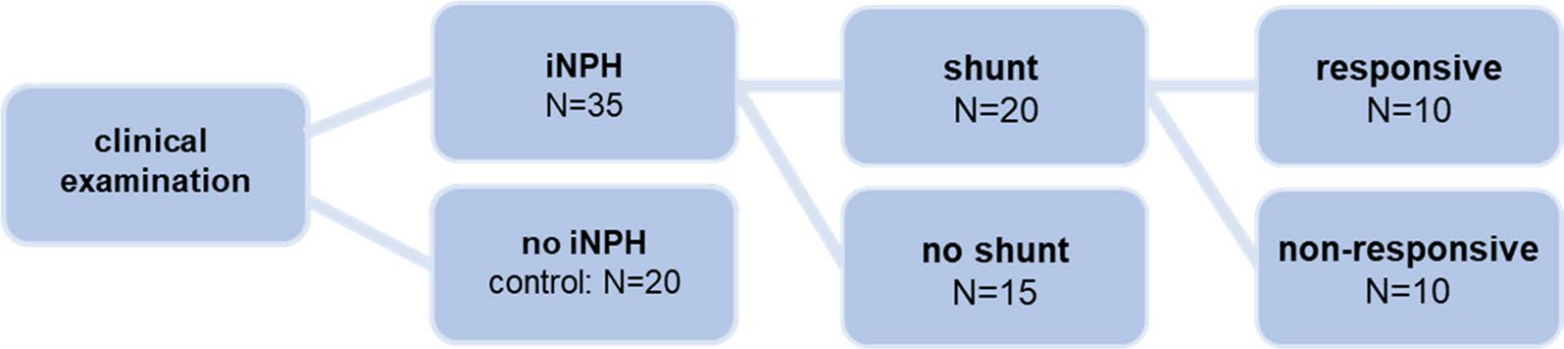
Patient groups. Upon clinical examination, patients were divided into those who fulfilled the diagnostic criteria for iNPH (iNPH) and those that did not (no iNPH). The latter group served as the control group. A group of iNPH patients were referred to shunt implant (shunt) and another was not (no shunt). Of the former group of shunted patients, some experienced relief of their symptoms at the follow-up examination (responsive) and some did not (non-responsive)

This study included CSF samples extracted from 35 iNPH patients. Patients were diagnosed with iNPH according to the international guidelines from 2005 [1], including evaluation of cognitive impairment, gait/balance disturbances, urinary incontinence and brain imaging. All patients had a supplementary diagnostic test (infusion test followed by a tap-test using the CELDA system (Likvor, Umeå, Sweden) through two lumbar needles). The infusion test measures resting intracranial pressure (ICP) and resistance to outflow (R_out_). An abnormally high R_out_ increases the diagnostic accuracy, but a normal R_out_ does not preclude iNPH [26]. The lumbar CSF sample was obtained from the infusion test during the diagnostic examination. Because of mild symptoms, 15 iNPH patients were not recommended shunt surgery (group: no shunt). Patients with mild symptoms had mild gait problems (mean gait score 3 = wide-based gait with sway, without foot corrections, on a gait scale from zero = normal gait and 8 = wheelchair bound [27]) and only mild cognitive dysfunction and/or mild urinary incontinence. iNPH patients in need of shunt surgeries had ventricular CSF collected upon insertion of the shunt 6.6 ± 8.3 months after the lumbar CSF sampling. A ventriculoperitoneal shunt (OSV 2 shunt) was placed intraventricularly through a frontal burr hole according to standard procedures at the Department of Neurosurgery at Rigshospitalet, Copenhagen, Denmark. Of these patients, ten responded positively to shunt surgeries (group: shunt responders) and ten did not benefit from the treatment (group: shunt non-responders), Fig. 1. Effect of shunting was evaluated objectively 3–12 months after shunting by experienced clinicians during an outpatient clinic visit. Shunt response was defined as significant improvement in at least one symptom without worsening of other symptoms. The shunt response evaluation has been described in detail in Carlsen et al. [28]. Gait was evaluated by a 10 m gait test, and scored on a 8 point gait scale [27], and cognition by Mini Mental State Examination (MMSE) and Addenbrooke’s Cognitive Examination (ACE) [29, 30], whereas urinary incontinence was evaluated subjectively [27]. Due to the retrospective design of the study, postoperative objective measures for gait and cognition were only available in approximately 75% of the patients, and in the remaining patients, scores were based on clinical evaluation at follow-up visit obtained from patient records. Twenty individuals, who were referred for evaluation on suspicion of cognitive dysfunction, but after evaluation had normal cognition and no neuroimaging or biomarkers suggesting organic brain disorder (including iNPH) were included as control group. The control and iNPH groups were not age and sex matched (control; mean age: 63.4 years, range 45–84 years, 9F/11M, iNPH no shunt; mean age: 72.2 years, range 69–89 years, 3F/12M, iNPH shunt responders; mean age: 73.9 years, range 66–79 years, 3F/7M, iNPH shunt non-responders; mean age: 72.4 years, range 57–82 years, 4F/6M), Table 1. Written informed consent was obtained for all patients and the study was approved by the Ethical committee of the Capital Region of Denmark (H-19001474 and H-18046630).

**Table 1:** Clinical characteristics of the study population

Characteristics	All iNPH patients	iNPH responders	iNPH non-responders	iNPH no shunt	Elderly control subjects
N	35	10	10	15	20
Age (mean, range)	75, 57–89 years	74, 66–79 years	72, 57–82 years	77, 69–89 years	63, 45–84 years
Sex (M/F)	25/10	7/3	6/4	12/3	11/9
MMSE (mean, range)	25, 11–30	22, 11–28	25, 19–29	28, 23–30	28, 25–30
ACE (mean, range)	75, 29–94	65, 29–85	73, 55–91	83, 58–94	NA
Gait score (mean, range)	3, 1–7	4, 2–7	3, 1–5	3, 2–4	NA
Urinary continence score (mean, range)	3, 1–5	3, 2–5	3, 1–5	2, 1–4	NA

Gait score: 1 (normal)—8 (wheelchair bound), urinary continence score: 1 (normal)—6 (bladder and bowel incontinence)

*iNPH* idiopathic normal pressure hydrocephalus, *MMSE* Mini-Mental State Examination: 0 (poor performance)—30 (optimal performance), *ACE* Addenbrooke’s Cognitive Examination: 0 (poor performance)—100 (optimal performance)

### Animals

Danish mixed breeds of Yorkshire, Danish Landrace and Duroc pigs (n = 17) with a mean weight of 24.02 ± 4.37 kg and an estimated age of 10–13 weeks were included. CSF collection was performed with the pigs placed in lateral recumbency under general anaesthesia. Anaesthesia was induced with an intravenous injection of propofol (*Propo Vet Multidose* 10 mg/ml, Zoetis, Finland, 1–3 mg/kg) and subsequently maintained with isoflurane (*IsoFlo Vet*, Zoetis, Finland), 1–2.5 vol% inhalation in a circle system after an intramuscular premedication with 1 ml/10 kg of a custom-made Zoletil 50 Vet-mixture [125 mg zolazepam, 125 mg tiletamine dry matter (*Zoletil50 Vet*, Virbac, Denmark) dissolved in 6.25 ml xylazine (20 mg/ml, *Rompun Vet*, Elanco, Denmark), 2.5 ml ketamine (50 mg/ml, *Ketaminol Vet*, MSD Animal Health, Denmark) and 2.5 ml butorphanol (10 mg/ml, *Torbugesic Vet*, Zoetis, Finland)]. Additional 0.07 ml acepromazine (10 mg/ml, *Plegicil Vet*, Pharmaxim, Sweden) and 0.5 ml methadone (10 mg/ml, Comfortan Vet., Dechra, Denmark) were administrated intramuscularly at the time of premedication. Upon shaving of all puncture sites and cleaning with 70% ethanol, 1 ml CSF was collected from cisterna magna and from the lumbar cistern (between L7 and S1) with 90 mm and 75 mm spinal needles, respectively. Both CSF samples were obtained with < 5 min interval approximately 3 h after induction of anaesthesia. Animals were excluded if puncturing failed at one site. To avoid bias, the order of CSF collection was randomly controlled for each animal. All animal experiments were performed on pigs employed for veterinary student training of abdominal surgical procedures and according to the legislation for animal protection and care, animal permission no. 2016-15-0201-00957 approved by the Danish Animal Experiments Inspectorate.

### Osmolality measurement

CSF samples from patients were centrifuged at 2000*g* for 10 min at 4°C within 2 h from collection, whereafter the supernatant was stored in 500 µl polypropylene tubes [31]. To prevent break down of solutes within the CSF prior to osmolality measurements, the samples were stored at – 80°C and care was taken to only thaw the samples the one time in association with the osmolality measurements. CSF samples from pigs were used for osmolality measurements directly after centrifugation. To prepare CSF samples for osmolality measurements, 100 μl of the CSF sample was transferred to osmometer Eppendorf tubes. The CSF osmolality was determined by a freezing point depression osmometer (Löser, Type 15, Berlin, Germany) with an accuracy of ± 1 mOsm.

### Statistical analysis

Data were tested for normal distribution using the Shapiro–Wilk test. Data obtained from experimental pigs were normally distributed and were analyzed with paired two-tailed *t*-test. Data obtained on human CSF did not follow normal distribution and therefore these were analyzed with Wilcoxon test (paired data) or Mann–Whitney test (unpaired data) to evaluate statistically significant differences between mean values of groups, as indicated in figure legends. Pearson’s correlation analysis was performed to determine correlations between two variables. P < 0.05 was considered statistically significant. Data are shown as mean values ± standard error of mean (SEM). GraphPad Prism (GraphPad Prism Software 9.0, Inc., La Jolla, CA, US) was applied for statistical analyses.

## Results

### No elevated osmolality in lumbar CSF obtained from iNPH patients

**Fig. 2:**
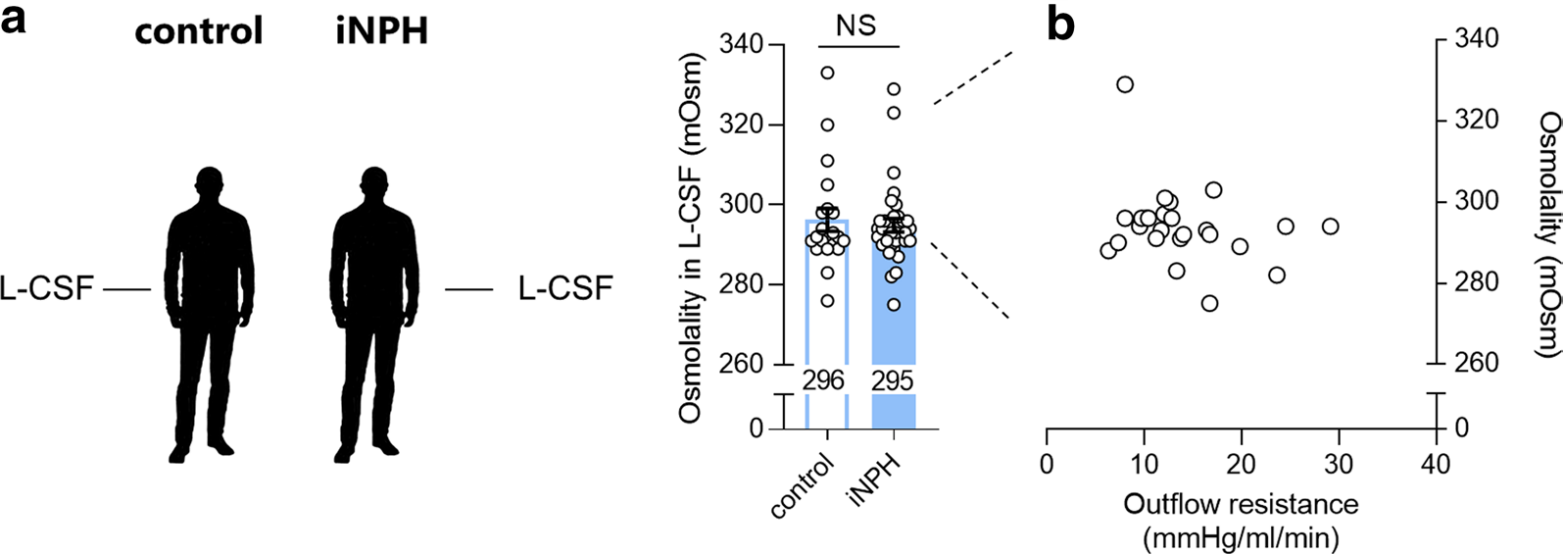
Osmolality of lumbar CSF obtained from iNPH patients versus control subjects. **a** The osmolality of lumbar CSF (L-CSF) in control subjects and iNPH patients, n = 20 control subjects and n = 35 iNPH patients. The data were evaluated for statistical significance with the Mann–Whitney test. **b** The osmolality of iNPH patient CSF as a function of the outflow resistance measured during diagnostic workup, n = 25 patients. *NS* not significant

To determine whether the enlarged ventricles observed in iNPH patients could develop subsequently to elevated osmotic pressure in the ventricular compartment, we compared CSF osmolality in patients with iNPH to that obtained from control subjects (Fig. 1). The lumbar CSF osmolality (296 ± 3 mOsm in control subjects, n = 20) was not significantly different from the lumbar CSF osmolality in iNPH patients (295 ± 2 mOsm, n = 35, P = 0.82, Fig. 2a). This finding suggests that the enlarged ventricles observed in iNPH patients are not a consequence of elevated osmotic pressure in the CSF with a subsequent increase in osmotic water flow into the ventricular compartments. The CSF osmolality in iNPH patients, in addition, displayed no correlation with the CSF outflow resistance (R_out,_ Pearson’s correlation coefficient (r) = − 0.28, n = 25, P = 0.17), Fig. 2b.

### iNPH patient CSF osmolality does not reflect the disease severity or shunt eligibility

**Fig. 3:**
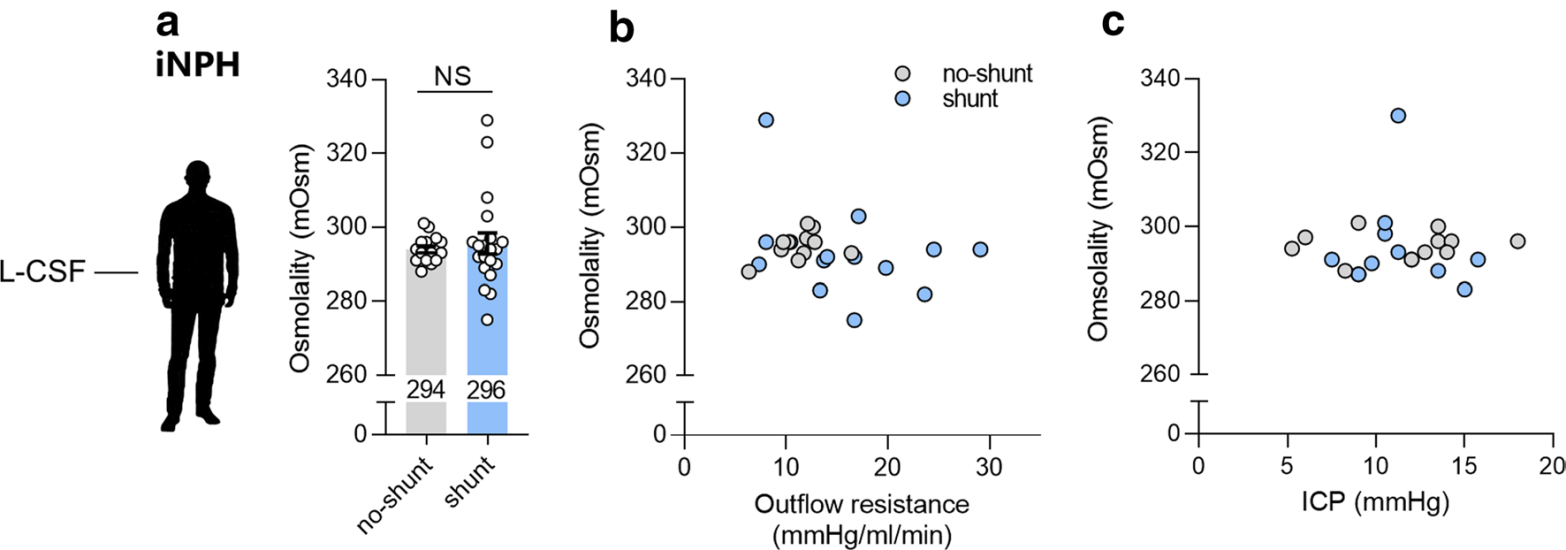
Osmolality of lumbar CSF obtained from iNPH patients selected for shunt surgery versus no surgery. **a** The osmolality of lumbar CSF (L-CSF) in iNPH patients not shunted (n = 15) versus those who were shunted (n = 20). The data were evaluated for statistical significance with the Mann–Whitney test. **b** The osmolality of iNPH patient CSF as a function of the outflow resistance measured during diagnostic workup, n = 11 shunted and 14 non-shunted patients. **c** Patient CSF osmolality plotted versus the resting ICP determined by lumbar infusion tests, n = 11 non-shunted patients and n = 12 shunted patients. *NS* not significant

To determine if CSF osmolality could be employed as a biomarker for disease severity and therefore for shunt eligibility, we evaluated the CSF osmolality in iNPH patients enrolled for shunt surgery versus those that were not (shunt vs. no shunt, see Fig. 1). The lumbar CSF osmolality of iNPH patients enrolled for shunt surgery (296 ± 3 mOsm, n = 20) was not statistically different (P = 0.86) from that of iNPH patients with no shunt referral (294 ± 1 mOsm, n = 15), Fig. 3a. The CSF osmolality of iNPH patients, therefore, seems not to reflect the disease severity.

The CSF outflow resistance in the iNPH shunt referral group (16 ± 2 mmHg/ml/min, n = 14) was significantly higher than that of the patient group not referred to shunt surgery (11 ± 1 mmHg/ml/min, n = 11, P < 0.05) reflecting that a high R_out_ was part of the decision to shunt immediately. The patient R_out_ did not correlate with the CSF osmolality in iNPH shunt (r = − 0.33, n = 14, P = 0.25) or no shunt (r = 0.40, n = 11, P = 0.23) patients, Fig. 3b. The resting ICP was similar for both patient groups (11.3 ± 1.5 mmHg for the non-shunted patients, n = 11 and 11.3 ± 0.7 mmHg for shunted patients, n = 12, P = 0.97) and displayed no correlation with CSF osmolality (r = 0.01, n = 11, P = 0.77 for non-shunted patients and r = 0.01, n = 12, P = 0.78 for shunted patients), Fig. 3c.

### Compatibility between CSF osmolality in lumbar and ventricular spaces

**Fig. 4:**
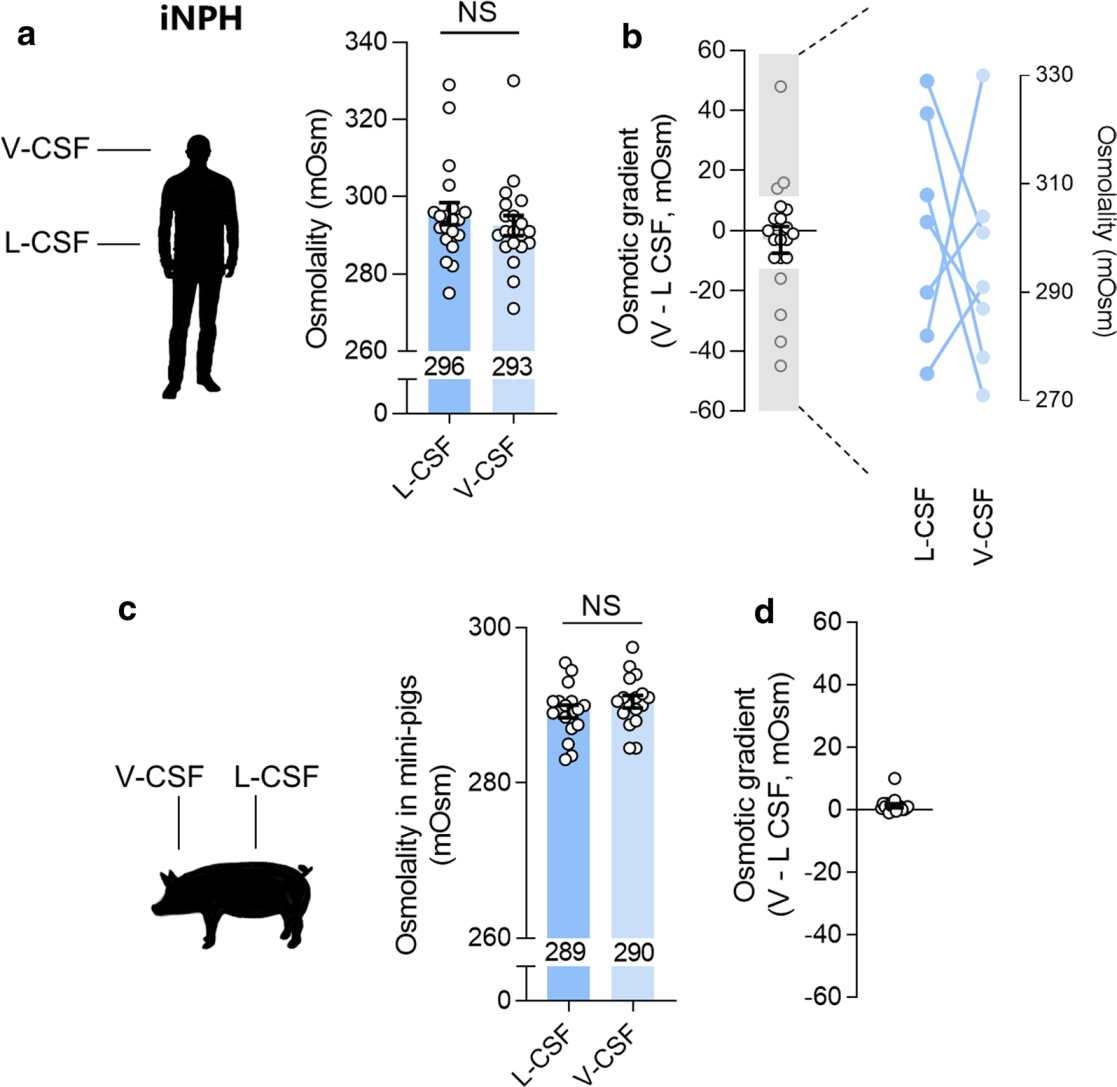
Osmolality of lumbar versus ventricular CSF. **a** The osmolality of lumbar CSF (L-CSF) versus ventricular CSF (V-CSF) in iNPH patients, n = 20. **b** The difference in lumbar versus ventricular osmolality in individual iNPH patients, with those with larger differences expanded in the right part of the panel. **c** The osmolality of lumbar CSF (L-CSF) versus ventricular CSF (V-CSF) in mini-pigs, n = 17. **d** The difference in lumbar versus ventricular osmolality in individual pigs, n = 17. The data were evaluated for statistical significance with the Wilcoxon test for the human data and with a paired *t*-test for the pig data. *NS* not significant

To determine the osmolality in paired lumbar and ventricular CSF samples from individual iNPH patients, we employed the lumbar CSF sample taken upon the diagnostic workup (from Fig. 3a; shunt group) and a subsequent ventricular CSF sample collected during the shunt implantation. The osmolality in lumbar CSF of the iNPH patients (296 ± 3 mOsm) was not significantly different from the osmolality in ventricular CSF (293 ± 3 mOsm), n = 20, P = 0.72, Fig. 4a. The ventricular CSF was 3 ± 4 mOsm lower than the individual lumbar equivalent, n = 20, Fig. 4b. However, a subset of patients (7/20) had differences > 10 mOsm in their ventricular versus lumbar CSF osmolality. As illustrated in Fig. 4b, these large differences occurred in both directions (ventricular-to-lumbar or lumbar-to-ventricular).

CSF samples such as those employed in this study are generally obtained ethically in connection with the relevant clinical procedures and therefore represent i) patient samples and ii) samples taken with  ~ 7 months interval representing the time between the diagnostic workup (lumbar samples) and the shunt surgery (ventricular samples). To obtain an experimental scenario in which we could determine the osmolality in both CSF compartments simultaneously in healthy organisms, we employed experimental pigs. Lumbar and ventricular CSF was collected from anaesthetized pigs at time points with only minutes in between. The osmolality of lumbar pig CSF was 289 ± 1 mOsm, which was not statistically different from the osmolality in ventricular CSF of 290 ± 1 mOsm (n = 17, P = 0.05, Fig. 4c). The intra-animal osmotic difference between the ventricular and lumbar spaces was 1 ± 1 mOsm (n = 17, Fig. 4d). These data suggest that the osmolality is similar in the lumbar and the ventricular CSF spaces on a group scale in both patients and experimental pigs.

### CSF osmolality is not a predictor of iNPH shunt surgery responsiveness

**Fig. 5:**
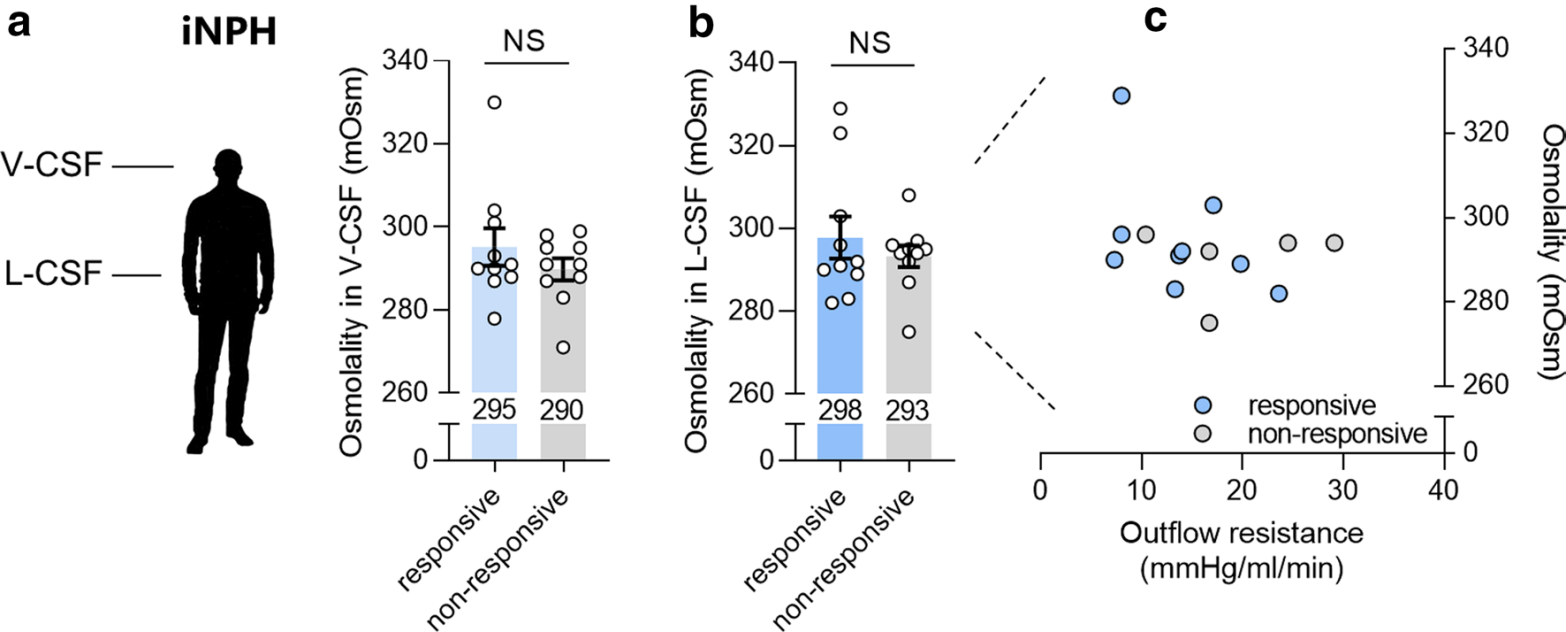
CSF Osmolality in shunt responsive versus non-responsive iNPH patients. **a** The osmolality of ventricular CSF in iNPH patients responsive to shunt surgery (n = 10) versus those that were non-responders (n = 10). **b** The osmolality of lumbar CSF in iNPH patients responsive to shunt surgery (n = 10) versus those that were non-responders (n = 10). The data were evaluated for statistical significance with the Mann–Whitney test. **c** The osmolality of iNPH patient lumbar CSF as a function of the outflow resistance measured during diagnostic workup, n = 10 of each patient group. *NS* not significant

To determine whether the responsiveness to shunt surgeries correlates with the CSF osmolality of iNPH patients, the patients were divided into either a shunt responsive or a non-responsive group at a follow-up evaluation of the treatment performed 3–12 months after the ventriculo-peritoneal shunt operation (see Fig. 1 for patient groups and Methods for clinical criteria). Depending on their clinical evaluation, the CSF osmolality from responsive iNPH patients (295 ± 5 mOsm, n = 10) was not significantly different (P = 0.67) from that of non-responsive iNPH patients (290 ± 3 mOsm, n = 10) in the ventricular compartment (Fig. 5a) or the lumbar compartment (responsive: 298 ± 5 mOsm vs non-responsive: 293 ± 3 mOsm, n = 10, P = 0.84, Fig. 5b). There was no correlation between the lumbar CSF osmolality and the outflow resistance in either of the patient groups (responsive: r = − 0.47, P = 0.20, n = 9; non-responsive: r = 0.16, P = 0.80, n = 5, Fig. 5c).

## Discussion

We here demonstrate that the enlarged ventricles characteristic of iNPH patients do not appear to arise due to an elevated osmolality of the patient CSF and that the shunt responsiveness does not hinge on the CSF osmolality.

Diagnostic workup of various patient groups employ CSF biomarker analysis. Although disturbed brain function is anticipated best reflected in the ventricular CSF bordering the affected brain regions, the CSF samples are generally obtained from the lumbar region. The lumbar sampling is swifter and less invasive than its ventricular counterpart and thus employed for ethical reasons and general feasibility. However, it remains unresolved to what extent the CSF composition at the base of the spine resembles that of the ventricular compartments. Blood-derived proteins, such as albumin, appear elevated in lumbar CSF [18, 19, 21], while the abundance of various neuroproteins may be constant [17, 21] or decline [20] towards the lumbar spinal region. Potential rostro-caudal CSF protein gradients may, however, vary with rate of CSF flow and clinical status of the patient [19, 32, 33]. We here show that the osmolality of CSF obtained from the lumbar region mirrors that of the CSF obtained from the ventricular compartment. The higher albumin content in lumbar CSF [18, 19, 21] contributes exceedingly little to the collective osmolality due to the low amounts (15–45 mg/100 ml) and high molecular weight (albumin; 66 kDa). Even with the slightly elevated albumin content in iNPH patients [34], the molar contribution of the protein content remains well below 20 μM (which approximates 20 μOsm of the 296 mOsm observed in lumber CSF samples, this study and [35]). The lumbar CSF samples were obtained during the initial diagnostic work up and the ventricular sample obtained with delay at the time of shunt implantation. Such a time gap was ethically required but represents a limitation to the study. However, a parallel experimental series on mini-pigs, in which we could obtain both samples within an interval of a few minutes, provided an identical dataset illustrating similar osmolality of the CSF obtained in the two compartments. Notably, the mini-pigs are animals of horizontal stature, versus the upright stature of humans, which could potentially represent a confounding effect. Altogether, our data suggest that the CSF osmolality remains stable throughout the CSF system.

The osmolality of bulk CSF is similar to that of the plasma [11], and CSF secretion thus appears to be able to occur in the absence of conventional osmotic forces and, curiously, can readily proceed even in the face of an experimentally-inflicted, oppositely-directed osmotic gradient [11–13]. Such fluid secretion is proposed to take place by a mechanism relying on transporter-mediated water transport [11, 36, 37]. However, the CSF secretion rate increases with elevated ventricular osmolality [11–14, 38], in the order of 0.4% elevation of the secretion rate with each milliosmole in the rat [11]. Notably, acute elevation of CSF osmolality with bolus administration of osmotic challenges up to ten-fold higher than that of the healthy rat provided only temporary ventriculomegaly, which was resolved 24 h later [15, 16]. However, continuous ventricular delivery of CSF with elevated osmolality (+ 30 mOsm) to experimental rats promoted ventriculomegaly [15, 16] and it was therefore speculated whether some forms of hydrocephalus could arise following a sustained elevation of CSF osmolality. We here demonstrate that CSF obtained from iNPH patients is of comparable osmolality to CSF obtained from control individuals. It should be noted that due to the retrospective nature of this study, there were age and sex differences between the groups and we cannot exclude that our findings may have been impacted by these differences. The patient CSF outflow resistance, when employed as a marker of disease pathophysiology, did not correlate with the patient CSF osmolality. The ventriculomegaly observed in iNPH patients therefore does not seem to arise as a consequence of elevated CSF osmolality and their CSF osmolality cannot be employed as a biomarker to detect iNPH development. A similar finding was earlier reported for idiopathic intracranial hypertension patients, the CSF osmolality of which resembled that of control individuals [39]. We cannot rule out that small elevations in CSF osmolality, which may have gone undetected in the present study, could suffice to cause a slight hypersecretion, which over time could promote the ventriculomegaly characteristic of iNPH.

Many iNPH patients, regrettably, do not benefit from shunt insertion [40]. To spare these patients from unnecessary neurosurgery, clinicians would welcome a biomarker indicative of potential shunt responsiveness [24]. In our search for a predictive marker with which to select iNPH patients for shunt surgery, we compared the osmolality of CSF obtained from patients who experienced relief of symptoms upon shunt placement versus those that did not. The CSF osmolality of these two patient groups were similar and CSF osmolality thus cannot be employed as such a marker of shunt responsiveness. Future quantification of protein markers obtained from shunt responders versus non-responders may deliver such a biomarker.

In conclusion, the CSF osmolality appears to be stable throughout the CSF system, as revealed by samples obtained in iNPH patients and in healthy pigs. Such stability allows lumbar CSF sampling to obtain osmolality determinations. iNPH patients do not present with elevated CSF osmolality and their ventriculomegaly, therefore, does not appear to arise as a function of osmotic imbalances in the CSF system. CSF osmolality thus cannot be employed as a clinical marker for disease progression or as a predictive tool for shunt responsiveness.

## Data Availability

Data are available upon reasonable request to the corresponding author.

## Abbreviations

CSF: Cerebrospinal fluid
ICP: Intracranial pressure
iNPH: Idiopathic normal pressure hydrocephalus
R_out_: Outflow resistance
